# How Accurate Can Crystal Structure Predictions Be for High-Energy Molecular Crystals?

**DOI:** 10.3390/molecules28114471

**Published:** 2023-05-31

**Authors:** Xavier Bidault, Santanu Chaudhuri

**Affiliations:** 1 Department of Civil, Materials and Environmental Engineering, University of Illinois at Chicago, Chicago, IL 60607, USA; xavbdlt@uic.edu; 2 Applied Materials Division, Argonne National Laboratory, Lemont, IL 60439, USA

**Keywords:** crystal structure prediction, high-energy molecular crystals, evolutionary algorithm, DFT-D

## Abstract

Molecular crystals have shallow potential energy landscapes, with multiple local minima separated by very small differences in total energy. Predicting molecular packing and molecular conformation in the crystal generally requires ab initio methods of high accuracy, especially when polymorphs are involved. We used dispersion-corrected density functional theory (DFT-D) to assess the capabilities of an evolutionary algorithm (EA) for the crystal structure prediction (CSP) of well-known but challenging high-energy molecular crystals (HMX, RDX, CL-20, and FOX-7). While providing the EA with the experimental conformation of the molecule quickly re-discovers the experimental packing, it is more realistic to start instead from a naïve, flat, or neutral initial conformation, which reflects the limited experimental knowledge we generally have in the computational design of molecular crystals. By doing so, and using fully flexible molecules in fully variable unit cells, we show that the experimental structures can be predicted in fewer than 20 generations. Nonetheless, one must be aware that some molecular crystals have naturally hindered evolutions, requiring as many attempts as there are space groups of interest to predict their structures, and some may require the accuracy of all-electron calculations to discriminate between closely ranked structures. To save resources in this computationally demanding process, we showed that a hybrid xTB/DFT-D approach could be considered in a subsequent study to push the limits of CSP beyond 200+ atoms and for cocrystals.

## 1. Introduction

The prediction of crystal structure for molecular crystals involves the use of highly accurate computational methods to determine the arrangement of molecules in the solid state. There are various approaches to crystal structure prediction [1], including ab initio methods, empirical force field models, and machine learning algorithms. The choice of method depends on the level of accuracy required to represent bonded and non-bonded interactions, the complexity of the crystal polymorphs in their respective potential energy surface, and the computational resources available. Empirical force field models use parameterized energy functions to describe the interactions between molecules, while machine learning algorithms make predictions based on patterns in training data. Regardless of the approach used, crystal structure prediction (CSP) requires a thorough understanding of the intra- and inter-molecular forces that determine the arrangement of molecules in the solid state, in their most stable form at finite temperatures. Due to subtle differences in synthesis methods, solvent-mediated stabilization of facets, and surface free energy effects, the crystallized form may not be the lowest energy structure. Thus, kinetic control through synthesis and crystallization conditions can also lead to different forms of crystals, making a better understanding of stable and metastable crystalline polymorphs equally important for pharmaceutical and other engineered molecular crystals. The accuracy of theoretical methods and the computational efficiency of CSP algorithms have improved over the past two decades with the increased availability of large parallel computing infrastructures. A large blind test [1] and related published work and benchmarks [2,3,4,5,6,7] show great advances in CSP for molecular crystals. The successes cover molecules of various sizes and various numbers of atoms in the unit cells, and account for molecular flexibility of up to eight internal degrees of freedom. Still, energetic molecular crystals are underrepresented in the literature [5,8,9]. To identify the challenges faced by commonly used theoretical methods and CSP algorithms, we investigate CSP capabilities for some known energetic molecular crystals. To represent the complexity of molecular building blocks and their diversity of interactions in the condensed phase, we explore the challenging crystal structure and potential energy landscape of 1,3,5,7-Tetranitro-1,3,5,7-tetrazocane (HMX), 1,3,5-Trinitro-1,3,5-triazinane (RDX), 2,4,6,8,10,12-Hexanitro-2,4,6,8,10,12-hexaazaisowurtzitane (CL-20), and 2,2-Dinitroethene-1,1-diamine (FOX-7) and their polymorphs. The insight gained will be valuable for the exploration of unknown energetic crystals and cocrystals.

Increasing molecular complexity introduces a conformational flexibility that is less straightforward to manage in CSP, leading to challenges in finding stable crystal packing. The papers above have shown that considering molecules as rigid building blocks can lead to improved efficiency and good predictions for small molecules. However, different conformers can pack very differently and form numerous polymorphs, only some of which may be observed experimentally. For some molecules, the potential energy landscape can be very shallow. In such cases, where differences in energy between conformers and packing motifs are extremely small, higher levels of theory and ab initio methods are recommended. The required accuracy adds to the computational cost, and various strategies have been developed to circumvent it. Some involve the prior optimization of the isolated molecule, searching for the most stable conformers, which are then kept rigid for the CSP algorithms [4,5,7,8]. Some involve a hybrid force field and ab initio approach [5,6,9], in which the force field (FF) can be developed for the molecular systems of interest, but may then lack transferability. In this approach, the initial guess from this FF is generally good, and a DFT-D stage improves the expected energy ranking of the predicted structures [5,6]. Some fragment-based and machine learning methods are also in development and are on a trajectory of improved accuracy [10,11].

**Figure 1:**
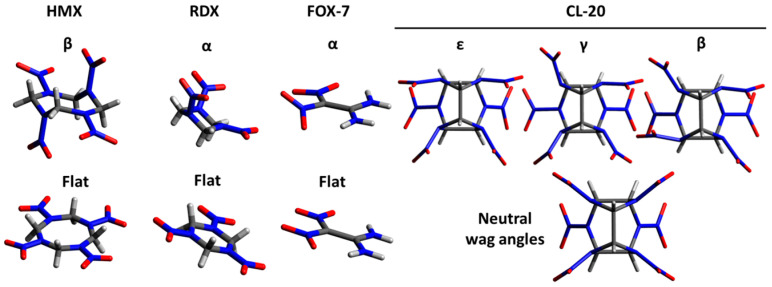
Molecules provided to USPEX, either in the conformation of the target crystal (top row) or in a flat/neutral conformation (bottom row).

With regard to the CSP of high-energy molecular crystals, Nikha et al. [5] included nitromethane (NM), 1,3,5-trinitrobenzene (TNB), and high-nitrogen 5,5′-dinitro-2H,2H′-3,3′-bi-1,2,4-triazole (DNBT) in their set. Small NM and benzenic TNB are not expected to have conformers, and although bi-triazolic DNTB could, they were all provided as rigid molecules to the CSP algorithm using a tailored FF. Despite the rigid approximation, the predicted structures of NM and DNTB agreed with the experimental ones, and that of TNB after a DFT-D stage. Wang et al. [8] also used a tailored FF to predict the polymorphs of CL-20, a cage molecule whose flexibility only stems from its six nitro groups (Figure 1). The molecules were also provided as rigid to the CSP algorithm, in the conformation of the target polymorph. Expectedly, the β- and γ-conformations yielded the experimentally known structures of β- and γ-polymorphs, respectively. However, the experimentally known ε-polymorph was not found as the lowest energy structure originating from the ε-conformation of the molecule. This underscores the high level of accuracy needed for such molecular crystals. Pakhnova et al. [9] tried flexible molecules in a hybrid ReaxFF/DFT-D approach. Some of the molecules of interest were challenging, such as PETN with its four flexible branches, HMX with its flexible ring and nitro groups (Figure 1), and CL-20 with its six flexible nitro groups. The layered structure of the TATB crystal was quickly and correctly predicted, as well as the crystal of PETN-I. The TNT molecule has very limited conformational possibilities. Despite that, the crystal of TNT was not found, and this was attributed to the higher number (eight) of molecules per unit cell. The crystal of ε-CL20 was not found either, but the selected ReaxFF parameterization was clearly not accurate enough for the case of CL-20 crystals. The crystal of β-HMX was only predicted if the CSP algorithm was supplied with the HMX molecule in β-conformation, a strategy that will be hard to transfer to unknown energetic crystals and cocrystals. Unfortunately, these deficiencies reduce the relevancy of the final part of the study about cocrystals. For instance, with regard to the CL-20:HMX cocrystals, if the 2:1 ratio and the CL20/CL20/HMX layered structure are correctly predicted [12], the orientation of the molecules and of the nitro groups is erroneous. A further prediction of properties such as the thermomechanical properties [13] or shock sensitivity [14] will be hard to initiate from these inexactly predicted structures. This serves as a good example of the downstream complexity and the high bar needed for accuracy in structure prediction before property predictions can be attempted. Often, density and lattice parameters are used as a metric, and such a simplistic approach can be problematic for the sensitivity prediction of polymorphs.

In the present work, we propose to properly assess the CSP capabilities for four differently challenging high-energy molecules: HMX, RDX, CL-20, and FOX-7 (Figure 1). To mention some of the challenges: these molecules have flexible nitro groups whose orientations can yield various molecular packings and known polymorphs with energies within a very narrow range; the HMX molecule has a ring structure allowing for boat or chair conformation; the targeted α-RDX crystal has eight RDX molecules per unit cell; and a variety of weak intra- and inter-molecular interactions is involved. In this work, in the entire CSP workflow, we consider the molecules with their full degree of flexibility leading to a large dimensionality for the conformer optimization space. In this less restrictive sandbox, we also assess how providing them in an initial flat or neutral conformation affects the prediction of the molecular crystal structures. For best accuracy, all the generated structures undergo variable-cell optimization using ab initio methods only, and a relevant hybrid approach is discussed at the end.

## 2. Methods

### 2.1. Molecular Crystal Structure Generation and Analysis with USPEX

The evolutionary algorithm (EA) embedded in USPEX 10.5.2 [15] (Universal Structure Predictor: Evolutionary Xtallography) was used to carry out structure prediction for molecular crystals. Due to the expensive ab initio methods used for variable-cell relaxation, we restricted the initial population and subsequent generations to 20 structures, plus a re-optimization of the 3 best structures at every generation. The initial structures were generated by USPEX using the same CHON distance cutoffs as in Pakhnova et al.’s study [9], and the cutoff distance between molecular centers was set to 4.0 Å, which is larger than the size of a half molecule. While every molecular crystal presently studied has one independent molecule per asymmetric unit (Z’ = 1), USPEX was provided with the actual Z molecules per unit cell as independent (the influence of Z is beyond the scope of the present work). The molecules were provided either in the conformation of the targeted crystals (from the respective cif files), or more challengingly in a neutral (and hopefully unbiased) conformation. To do so, the molecules of interest were simply drawn from scratch in Avogadro [16] in a flat configuration (or 3D for CL-20) and slightly optimized using the UFF force field—just a few steps to obtain reasonable bond distances and angles. The resulting conformations are displayed in Figure 1. Then, the generated structures were optimized using the ab initio methods described hereafter, with no enforced symmetry, and with no constraint on the lattice parameters or on the molecules, which were all totally flexible. The resulting relaxed structures were then analyzed by USPEX and ranked from the lowest energy. Evolutions were considered up to 20 generations, involving heredity, random generation from specified space groups (SGs), soft mutation, rotational mutation, and lattice mutation (more details can be found in [2]), for which various fractions have been tested, as specified for every molecule tested hereafter. From our observation of the structures found by Pakhnova et al. [9], we deemed it relevant to enhance the fraction of rotational mutation, presently set to 0.4 in our simulations, to allow for more opportunities to modify the orientation of the molecules in the crystal, and of their nitro groups. The maximum degree of mutation in soft mutation and lattice mutation was set to 4.0 Å. The hereafter so-called “common space groups” for molecular crystals were selected to include SGs 14, 2, 19, 15, 4, 61, 29, 33, 7, 9, and 62 [2,10,17], complemented with rare SGs 43 (α-HMX) and 114 (PETN-I). To convert energy into eV, USPEX uses the factors 1 Ry = 13.6056923 eV for Quantum Espresso and 1 Ha = 27.211385 eV for CP2K. To assess the similitude between the best USPEX structures and the references (experimental structures optimized as in Section B), we compared the lattice parameters, SGs, energy, and molecular conformations. Along with the visual inspection, the structural similitude was further quantified using the normalized dot product (denoted CxC) of the total radial distribution function (RDF) of both the USPEX and reference structures. The closer to 1, the better the similitude. This CxC index is implemented in the MAISE package [18], and we used the default parameters (R_soft_ = 5.8 Å, R_hard_ = 6.0 Å, and a Gaussian broadening σ = 0.008 Å). Input files for HMX Simulations 1b, 5b, 7, and 8b (Table 1) are provided in the Supplementary Materials.

### 2.2. Ab Initio Variable-Cell Optimizations

Ab initio variable-cell relaxations were carried out using the density functional theory (DFT) as implemented in Quantum Espresso 6.7 (QE) [19] or CP2K 9.1 [20], with the Perdew–Burke–Ernzerhof (PBE) generalized gradient approximation (GGA) functional, and the Grimme D2 correction (or D3(BJ) for all-electron calculations) for a proper description of the vdW interactions, generically denoted PBE-D. Details relative to QE and CP2K are described hereafter.

#### 2.2.1. Quantum Espresso

Featured for high-throughput DFT calculations, we considered version 1.5 of the Garrity–Bennett–Rabe–Vanderbilt non-linear-core-corrected ultrasoft plane-wave pseudopotential (GBRV NLCC US PP). An energy cutoff of 50 Ry for wave functions (and 500 Ry for charge density) was found satisfactory to balance the simulation time and accuracy (asymptotic error < 10 meV/atom). The default value of 0.7 for the mixing factor and a convergence threshold of 10^−6^ Ry were used for self-consistent field (SCF) calculations.

References for the molecular structures of interest were obtained through variable-cell relaxation. The convergence was monitored through the Broyden–Fletcher–Goldfarb–Shanno (BFGS) algorithm for both lattice parameters and atom positions, using thresholds of 10^−5^ Ry for the system energy, 10^−4^ Ry/Bohr for the forces, and 0.1 kbar for the pressure tensor (which is diagonal and isotropic once the configuration is relaxed). The Brillouin zone was sampled using a 3 × 2 × 3 Monkhorst–Pack off-grid k-point mesh for β-HMX, 2 × 2 × 2 for α-RDX, 3 × 3 × 2 for α-FOX-7, and 2 × 2 × 2 for ε-CL20. The resulting lattice parameters are in excellent agreement with ambient experimental data.

For time efficiency, the convergence parameters for variable-cell relaxation of the USPEX-generated structures are less stringent: 10^−4^ Ry for the system energy and 10^−3^ Ry/Bohr for the forces, with a maximum of 250 steps. A “k-point resolution” value of 0.08 in USPEX automatically generates an on-grid k-point mesh for QE, avoiding simulations restricted to the mere Γ-point. No symmetry was enforced on k-points, lattice parameters, or atomic positions.

#### 2.2.2. CP2K

For the hybrid Gaussian and plane waves approach of CP2K, we considered the GTH-NLCC PP (Goedecker–Teter–Hutter) with the molecularly optimized (molopt) basis set mDZVP-SR (SR stands for short range). The energy cutoff of 600 Ry was found to yield excellent accuracy (asymptotic error = 0.02 meV/atom). The default value of 0.4 for the mixing factor and a convergence threshold of 10^−6^ Ha were used for SCF calculations, although we used a more stringent value of 10^−8^ Ha for FOX-7. For FOX-7 and CL-20, we considered all-electron (AE) calculations with the polarized triple-ζ 6-311G** basis set along with Grimme D3(BJ) dispersion. We also considered a lower level of theory, the extended tight-binding xTB method as implemented in the latest CP2K update (git:d529ce5). The GFN1-xTB [21] method includes the D3(BJ) dispersion correction, and we used the default value of 10^−3^ for the COULOMB_SR_EPS parameter, the same convergence threshold as above, but a mixing factor of 0.3.

References for the molecular structures of interest were obtained the same way as with QE, using thresholds of 10^−4^ Ha for the system energy, and 10^−5^ Ha/Bohr for the forces. The resulting lattice parameters are in excellent agreement with ambient experimental data. Less stringent parameters were used for the USPEX-generated structures, of 10^−4^ Ha for the system energy and 10^−3^ Ha/Bohr for the forces, with a maximum of 250/500/500 steps for the PP/AE/xTB simulations, respectively. USPEX does not generate a k-point mesh for CP2K, so a 2 × 2 × 2 on-grid k-point mesh was used for structures involving HMX, RDX, and CL20 structures, and 3 × 3 × 3 for FOX-7 structures. No symmetry was enforced on k-points, lattice parameters, or atomic positions.

## 3. The β-HMX Test Case

In Section 3, Section 4, Section 5 and Section 6, we present and discuss the structures that USPEX found using QE or CP2K with PP and AE calculations. Then, in Section 7, we assess the relevancy of the cheaper but less accurate xTB method.

The crystal of β-HMX (CCDC ref. 792930), in SG 14 (P2_1_/n), is the stable form at ambient conditions. It contains two molecules of HMX (C_4_H_8_O_8_N_8_) per unit cell. The USPEX parameters for the HMX simulations are reported in Table 1, whose last column presents the generation at which β-HMX was found. Table 2 reports the energy and lattice parameters of the QE- and CP2K-optimized crystals, which represent the references to which the best structures found by USPEX are compared.

### 3.1. Importance of the Good First Guess

Given the composition of the HMX molecule, USPEX sets the default volume of the crystal at 432 Å^3^ and generates the structures with this initial constraint. This is 15% lower than the reference volume (Table 2) and relates to Simulations 1a and 1b in Table 1, where the HMX molecules are initially in the β-conformation. In Simulation 1a, USPEX randomly generates structures only from SG 14, and β-HMX was found at the first generation. In Simulation 1b, randomly generating from the common SGs does not take much more time to find β-HMX as well. The expected structure was refined at the second generation, from an excellent guess at the first one. It seems that this tight initial volume favors the discovery of the packing matching the given molecular conformation.

**Figure 2:**
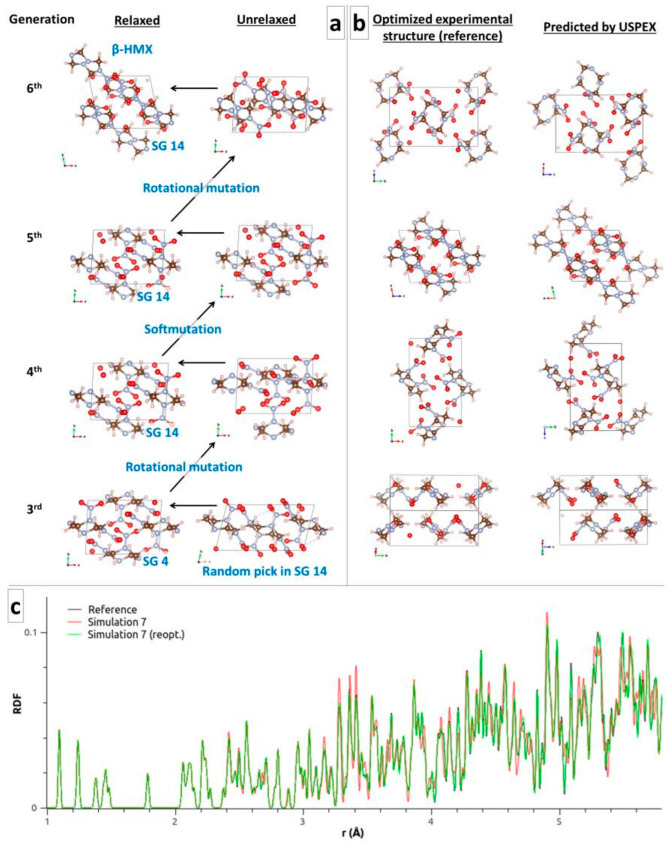
(**a**) Evolution leading to β-HMX in Simulation 2a. (**b**) Visual comparison of the reference and USPEX structures of β-HMX (Simulation 7). The “P2_1_/a” lattice parameters from USPEX were switched to “P2_1_/n” and the axes were permutated for a straightforward comparison in Table 2. The conformation of the molecules as well as their relative positions and orientations are in excellent agreement, with a similitude index CxC of 0.942, as obtained from the RDFs in (**c**). Further optimization of the USPEX structure with a force tolerance of 10^−5^ Ha/Bohr yields an improved CxC of 0.998.

Pakhnova et al. [9] recommended taking an initial volume 20–30% larger than the USPEX default. Testing the upper range and fixing the initial volume to 570 Å^3^, it appears that many more generations (6–7 times more) are needed to find β-HMX. This setting offers too much space and too many possibilities, and the EA becomes useful. Starting from SG14 only (Simulation 2a), a random pick at the third generation sequentially undergoes a rotational mutation, a soft mutation, and another rotational mutation to yield β-HMX at the sixth generation (Figure 2a). Starting from the common SGs, β-HMX was found at the 14th generation, albeit from an excellent pick in SG 14 at the 13th generation. Therefore, getting a good first guess early in the process seems crucial, as observed throughout our study hereafter and for every molecule.

### 3.2. Rotational Mutation and Soft Mutation as the Most Relevant Evolutions

In Simulations 3a, 3b, and 3c, random structures were generated only at the first generation and from SG 4 only, in order to prevent the initial good guess. Further random picks were disallowed in the subsequent generations, where only heredity, rotational mutation, soft mutation, and lattice mutation were allowed. It turned out that rotational mutation was the most relevant evolution to quickly find β-HMX. Soft mutation mainly acted as an additional relaxation stage. Heredity and lattice mutation were less relevant because they do not change the orientation of the molecules in the crystal or of their nitro groups.

### 3.3. Neutral vs. β-Conformation of the Input Molecule

Even though the molecules in this work are totally flexible, providing a specific input conformation can favor the initial packing generated by USPEX (see Simulations 1a and 1b). In a computational design framework, the preferred conformation(s) of a newly-discovered molecule in a crystal may be a priori unknown. Therefore, in this section, we investigate the effects of providing the molecule in a naive, flat configuration. The initial volume is set to 520 Å^3^, which is between the tight default estimate of USPEX and the loose large one. In Simulations 4a and 4b, the HMX molecule is provided in β-conformation, whereas in Simulations 5a and 5b it is provided in flat conformation (Figure 1). Note that β-HMX was found in every simulation.

β-HMX was found in Simulations 4a and 4b within a number of generations between those of Simulations 1a and 1b (tight initial volume) and of Simulations 2a and 2b (loose initial volume), which confirms the trend already observed. β-HMX was also found from the initially flat molecules in Simulations 5a and 5b, although fewer generations were needed when starting from the common SGs than when starting from SG14 only. Actually, in Simulation 5a, β-HMX was found after a rotational mutation of a good random guess at the 12th generation.

The simulations using QE or CP2K yield similar results (see Simulations 4a vs. 6a, 4b vs. 6b, and 5b vs. 7). β-HMX is found quickly and further refined. Note that β-HMX found at the 12th generation of Simulation 6b comes from a random guess in SG 7 at the 1st generation, which undergoes 7 rotational mutations and 4 in-between generations as “kept best”. This kind of evolution favorably compensates for the low rate of random picks in SG 14, representing less than 10% of all the picks in the common SGs. Unfortunately, these evolutions do not always succeed in transforming a pick in a random SG to the targeted SG and structure (see CL-20 hereafter).

Figure 2b displays a side-by-side comparison of selected orientations of the reference structure of β-HMX to the structure predicted by USPEX in Simulation 7 (involving the common SGs and initially flat molecules). The conformation of the molecules as well as their relative positions and orientations are in excellent agreement, with a similitude index CxC of 0.942, as obtained from the RDFs in Figure 2c. The black (reference) and red (Simulation 7) curves show tiny structural differences beyond 3 Å. Further optimization of the USPEX structure with a force tolerance of 10^−5^ Ha/Bohr yields an improved RDF (green curve in Figure 2c) and an excellent CxC of 0.998. However, the energy improvement is tiny, going from −0.04 meV/atom to −0.00 meV/atom with respect to the reference, meaning that the force tolerance of 10^−3^ Ha/Bohr for CSP is enough, even though the atomic positions and lattice parameters do not perfectly match the reference.

## 4. Handling More Molecules and (Re)Discovering α-RDX

The RDX molecule (C_3_H_6_O_6_N_6_) is also ring-like, but smaller than HMX. The crystal of α-RDX (CCDC ref. 1131953), in SG 61 (Pbca), is the stable form at ambient conditions. The challenge here is that the unit cell contains eight molecules with different relative orientations. Table 3 reports the USPEX parameters for the various simulations and the generation at which α-RDX was found.

**Figure 3:**
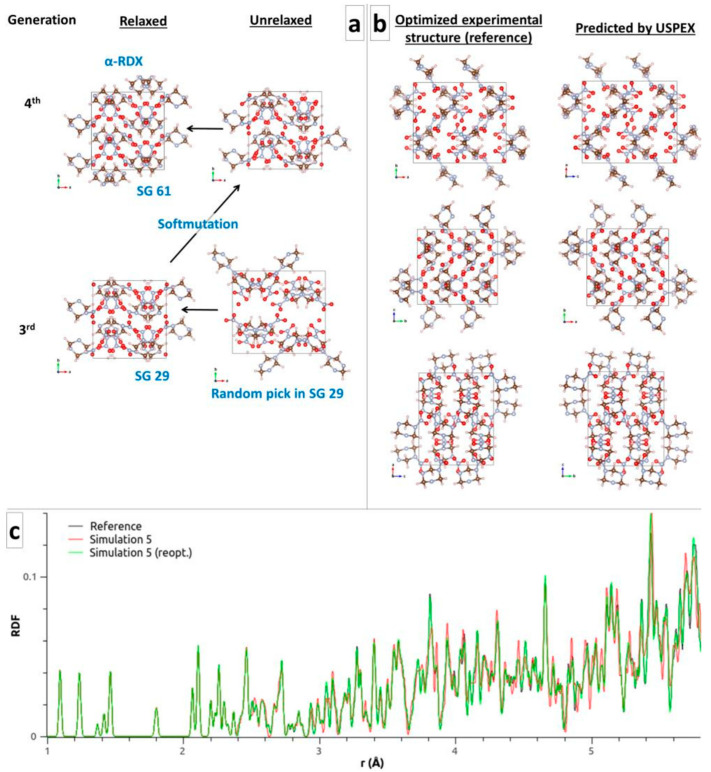
(**a**) Evolution leading to α-RDX in Simulation 3. (**b**) Visual comparison of the reference and USPEX structures of α-RDX (Simulation 5). The axes were permutated for a straightforward comparison of the lattice parameters in Table 4. The conformation of the molecules as well as their relative positions and orientations are in excellent agreement, with a similitude index CxC of 0.928, as obtained from the RDFs in (**c**). Further optimization of the USPEX structure with a force tolerance of 10^−5^ Ha/Bohr yields an improved CxC of 0.996.

Starting from RDX in α-conformation and SG 61, α-RDX was found at the first generation, using the default volume estimated by USPEX (Simulation 1a) or a volume 25% larger (Simulation 1b). Starting from the common SGs again did not take much longer, where α-RDX was found at the third generation after two rotational mutations of a random pick in SG 61 (Simulation 2a) and at the first generation (Simulation 2b). Adding complexity and starting from the flat molecule (Simulation 3) was also a success, where the α-RDX structure found at the fourth generation came after the soft mutation of a random pick in SG 29 at the third generation (Figure 3a). Once again, simulations using QE or CP2K yield similar results (see Simulations 2b vs. 4 and 3 vs. 5). Table 4 shows the energy and lattice parameters of the best structures obtained from the most naive simulations (Simulations 3 and 5), starting from the flat conformation of the molecule and the common SGs. They compare very well to the calculated references. The α-RDX structure predicted in Simulation 5 compares well visually to the optimized reference (Figure 3b). The conformation of the molecules as well as their relative positions and orientations are in excellent agreement, with a similitude index CxC of 0.928. Further optimization with a force tolerance of 10^−5^ Ha/Bohr yields a CxC of 0.996.

## 5. The Shallow Conformational Differences and Hindered Evolutions of CL-20

The challenge for the cage-like CL-20 molecule (C_6_H_6_O_12_N_12_) comes from the range of possibilities and combinations for the orientation of its six nitro groups and for the relative orientations of the molecules. The larger the molecule, the more possibilities there are [22,23,24]. Energy variations due to the conformational changes can be very small and many structures can lie within 1 meV/atom or less. Moreover, the naturally slow, sluggish, or one-directional phase transformations [25] can give a glimpse of some of the difficulties of structure-to-structure evolutions. Four polymorphs are known at ambient conditions, although γ-CL20 is probably the stable form, and ε-, β- and α-CL20 are metastable [26], the latter being the preferred form at high temperatures. Here, we focus on ε-, γ-, and β-CL20 (CCDC ref. 117779, 117778, and 117777, respectively), which have four molecules per unit cell in common. ε-CL20 and γ-CL20 crystallize in SG 14 (P2_1_/n), and β-CL20 in SG 29 (Pb2_1_a), with the respective conformations shown in Figure 1. Table 5 shows how QE and CP2K rank these polymorphs. It would be expected that USPEX with QE yields ε-CL20, and more neatly with CP2K using AE simulations. However, it would be expected that CP2K using PPs yields γ-CL20, but by a tiny margin. The USPEX parameters for CL-20 simulations are reported in Table 6. All the simulations started with CL-20 molecules in the neutral conformation displayed in Figure 1. Note that γ-CL20 was never found as the best structure. Consequently, the last column indicates the generation at which ε-CL20 was found, when relevant.

**Figure 4:**
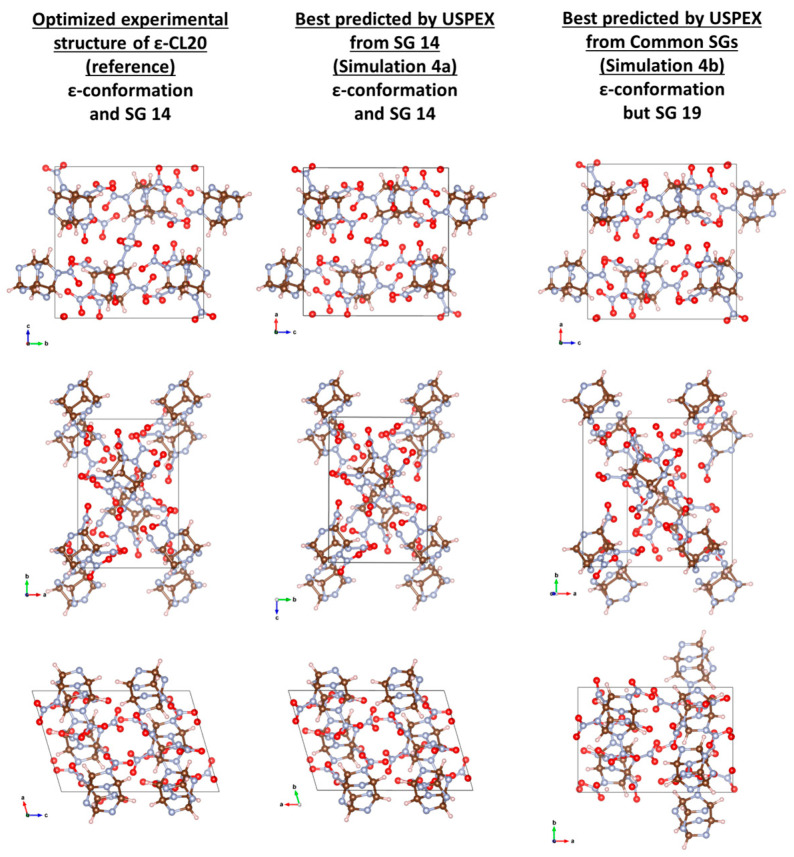
Visual comparison of the reference of ε-CL20 with USPEX best predictions when starting from SG 14 (Simulation 4a) or from the common SGs (Simulation 4b). For the former, the conformation of the molecules as well as their relative positions and orientations are in excellent agreement, with CxC = 0.969. For the latter, the molecules are in ε-conformation as expected, but packed in SG 19. Despite strong visual similarities, the structure in SG 19 is 0.87 meV/atom above ε-CL20, with a CxC of only 0.749 between both.

Starting from the volume estimated by USPEX and randomly picking in SG 14, ε-CL20 was found at the 15th generation from a random pick at the 2nd generation undergoing 5 rotational mutations, 1 soft mutation, and 7 in-between generations as “kept best” (Simulation 1). Starting from a volume 20% larger, ε-CL20 was found at the second generation after the soft mutation of a random pick at the first generation (Simulation 2a), or at the eighth generation from a good random pick (Simulation 4a—Figure 4), or even at the first generation in the AE simulation (Simulation 7a). However, the discovery of ε-CL20 fails when the search is expanded to the common SGs (Simulations 2b, 4b, and 7b). After 20 generations, all the structures generated and refined by USPEX are of higher energy than that of ε-CL20. The process could be considered still ongoing or incomplete. Interestingly, whether using QE and CP2K (PP or AE), the simulations are stuck in the same structure in SG 19, with only 0.92 meV/atom (QE), 0.87 meV/atom (CP2K PP), and 0.92 meV/atom (CP2K AE) above ε-CL20. The molecules are in the expected ε-conformation, but the β-angle mainly differs, yielding CxC = 0.749 (Figure 4). This structure is not closer to γ- and β-CL20, with CxC = 0.731 and 0.736, respectively. Those “best” structures in SG 19 have been used as seeds to which USPEX applied mutations only in Simulations 3 and 5. Unfortunately, after 10 generations, none of the mutations was able to make them evolve toward a structure of lower energy. Boosting the lattice mutation of the lattice angles up to a maximum of 10° did not solve the problem, where the angular mutation rather triggered a change in the conformation of the molecule in Simulation 5 (ε- to γ-conformation), resulting in structures of higher energy (hence not γ-CL20).

Technically, these “best” structures in SG 19 are pitfalls that prevent any potential good guesses in any relevant SGs to be kept and further refined. For instance, the good guess at the first generation of Simulation 2a is 3.47 meV/atom above this “best” structure. Unfortunately, after the 20 generations of Simulation 2b, USPEX kept only a few good structures within 1 meV/atom above this “best” one. So, even though a relevant good guess showed up at this point, it would be discarded by the algorithm and never refined. Only CP2K may stand a chance because the good random pick at the eighth generation of Simulation 4a is 0.80 meV/atom below the SG 19 “best” structure of Simulation 4b. However, statistically, with a pool of SGs 10 times larger than a single SG 14, one could expect to wait for about 80 generations to get this good random guess. In an attempt to circumvent this reasonable requirement, we have reduced the pool to solely SGs 14 and 19 in Simulations 6 and 8. Simulation 8 was stuck as of the second generation in the “best” structure of SG 19, 0.92 meV/atom above ε-CL20. In Simulation 6, USPEX found another “best” structure in SG 7 where the molecules are in β-conformation, only 0.09 meV/atom above ε-CL20, with CxC = 0.764. Even in this supposedly easier case, more generations are probably needed. Note however that USPEX did not maintain the balance between the two SGs, with only 36% of the structures randomly picked in SG 14 against 67% in SG 19 (and a 40/60 ratio in Simulation 8). The reason for this imbalance is unclear. We supposed that after a given number of failed attempts to generate in SG 14, USPEX switched to SG 19.

The difficulties encountered by USPEX in finding the lowest energy structure when randomly picking in the common SGs or simultaneously in SGs 14 and 19 is problematic. The antiseed technique has been tried with recommended parameters, but it appeared that 20 generations are not enough to get out of the “best” structure in SG 19. The imbalanced picking rate is another issue. Currently, one solution to overcome these pitfalls would be to run as many USPEX simulations as there are SGs of interest, one USPEX simulation per SG. Table 6 reports the properties of the calculated references and of the best structures found by USPEX. For each PBE-D level of theory presently tested, the ε-CL20 structures from USPEX are in excellent agreement with the ε-CL20 structures of reference (see also Figure 4 for Simulation 4a, Table 7).

## 6. The Case of Small but Not Easier FOX-7

The small molecule of FOX-7 (C_2_H_4_N_4_O_4_) is of deceitful simplicity. It crystallizes at ambient conditions as α-FOX-7 (CCDC ref. 616838), in SG 14 (P2_1_/n). The molecules form wave-shaped layers stacked along the “b” axis. This challenging structure comes from the network of strong intra- and inter-molecular hydrogen bonds contrasting with the weak inter-layer van der Waals interactions [27]. A moderate temperature yields β-FOX-7 (CCDC ref. 616841), in SG 19 (P2_1_2_1_2_1_) structurally very close to α-FOX-7 [27]. Table 8 shows that the energy differences between both are small. Only AE simulations rank them unambiguously. The USPEX parameters for FOX-7 simulations are reported in Table 9, where the last column displays the generation at which α, β, or another “best” polymorph was found. Their energies and lattice parameters are compared to the calculated references in Table 10.

**Figure 5:**
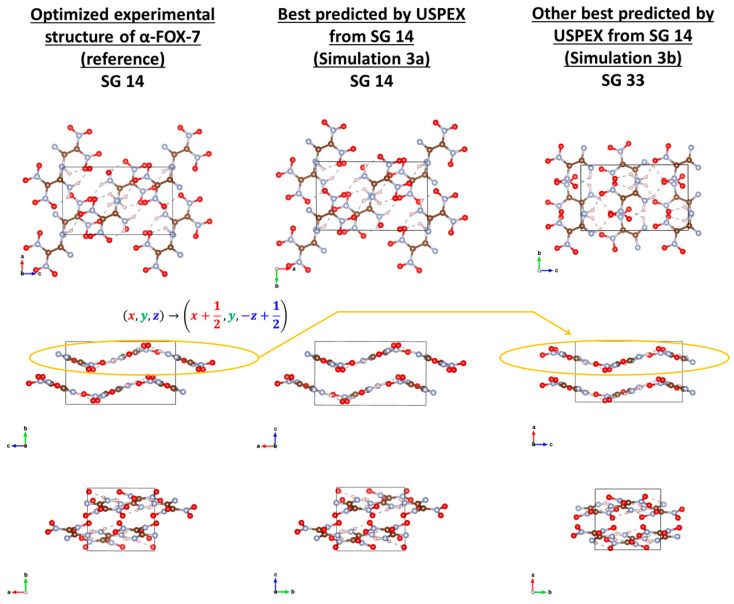
Visual comparison of the reference of α-FOX-7 with USPEX best predictions when starting from SG 14 (Simulation 3a and 3b). Simulation 3a found α-FOX-7 with a similitude index CxC of 0.832, which increases to 0.989 upon further optimization with a force tolerance of 10^−5^ Ha/Bohr. Simulation 3b found a wavy structure of the same energy (Table 10C.) but in SG 33. This structure in SG 33 can be obtained from α-FOX-7 by applying the transformation $\left(x,\;y,\;z\right)\to \left(x+\frac{1}{2},\;y,-z+\frac{1}{2}\right)$ to the top layer of the reference. This structure was also found in Simulations 3c and 1a.

**Figure 6:**
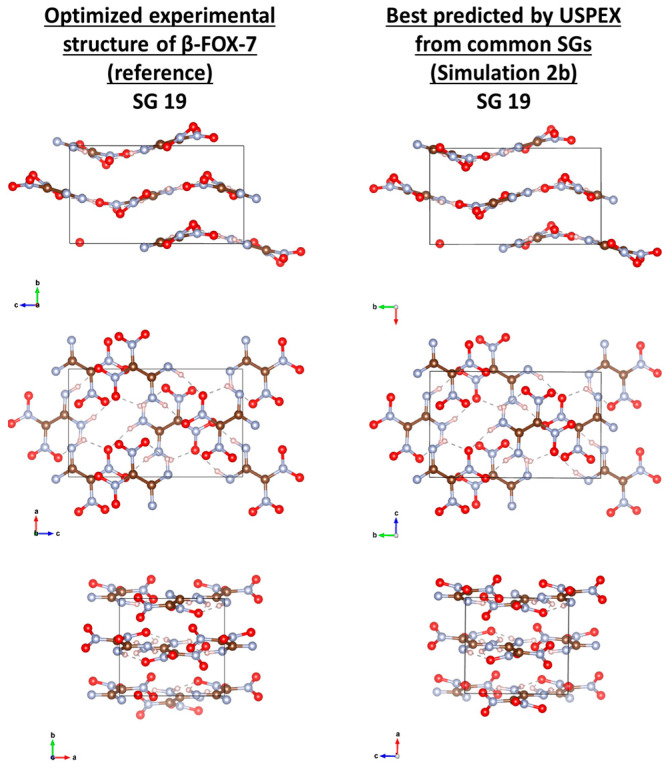
Visual comparison of the reference of β-FOX-7 with the structure predicted by USPEX when starting from the common SGs (Simulation 2b). The similitude index CxC is 0.996.

Despite starting only from SG 14, USPEX using PP methods with QE and CP2K did not find α-FOX-7 (Simulations 1a and 2a). Simulation 1a yielded its best structure in SG 33 instead (Figure 5, right column), with expected wavy layers, but with layer-to-layer orientation differing from that of α-FOX-7, and very close in energy (Table 10A). In Simulation 2a, a “best” structure was found in SG 14 but of significantly higher energy, and with its molecular layers planar and perpendicular to the wavy ones in α-FOX-7. Starting from the common SGs, β-FOX-7 was quickly and expectedly found in Simulations 1b and 2b (Figure 6), both after one rotational mutation of a good random pick in SG 19 at the previous generation. α-FOX-7 was found only in AE Simulations, at the sixth generation after the soft mutation of a good random guess at the previous generation (Simulation 3a; Figure 5, middle column), or from a good pick in SG 14 at the fifth generation (Simulation 3d). Interestingly, Simulations 3b (similar to 3a) and 3c (similar to 3d) yielded the same best structure in SG 33 as in Simulation 1a (Figure 5, right column), and again with its energy equal to that of α-FOX-7 (Table 10C). Actually, this structure in SG 33 can be obtained from α-FOX-7 by applying the transformation $\left(x,\;y,\;z\right)\to \left(x+\frac{1}{2},\;y,-z+\frac{1}{2}\right)$ to the top layer of the reference structure (Figure 5).

## 7. Can We Afford Faster Methods Than DFT-D?

### 7.1. Semi-Empirical GFN1-xTB

**Figure 7:**
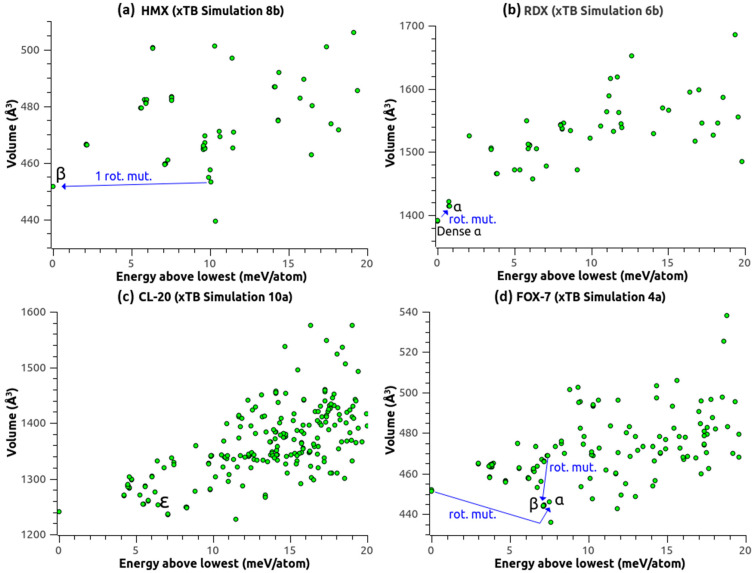
Volume vs. energy distributions of the structures predicted by USPEX using xTB. (**a**) β-HMX was found after the rotational mutation of a parent 10 meV/atom higher. (**b**) α-RDX came from the rotational mutation of the lowest energy structure. (**c**) ε-CL20 appeared at the 18th generation, too late and too high in energy to be retained in the best or good structures. (**d**) α-FOX-7 and β-FOX-7 were found as well, with α-FOX-7 coming from the rotational mutation of the lowest energy structure.

The DFT-D methods used above were accurate enough for USPEX to find the expected structures of the four molecular crystals presently studied, but they required a significant amount of computer resources (Table 11). May a less accurate but faster method be relevant when the expected and predicted structures are separated by much less than 1 meV/atom? The reactive and polarizable force field ReaxFF was devised to be 100–1000 times faster than DFT-D, but Pakhnova et al. [9] showed that the energy correlation between ReaxFF and DFT-D was not accurate enough for CSP. Note that the parameterization they used (not provided), as well as the ReaxFF-lg parameterization [28], does not preserve the structure of ε-CL20 (significantly wrong β-angle [9,29], among other deficiencies). In their hybrid ReaxFF/DFT-D approach, the final DFT-D optimization stage did not compensate for the USPEX/ReaxFF failure to predict the experimental structures of CL-20 [9]. Perhaps in the near future a new ReaxFF such as the neural network reactive force field [30] or a relevant parameterization of the DFTB-ChIMES model [31] may fulfill the requirements of high transferability and accurate energy scaling. In the meantime, we put to the test a less approximate level of theory: the semi-empirical and extended tight binding (xTB) method GFN1-xTB [21], as implemented in the latest version of CP2K. It can be seen from Table 2, Table 4, Table 7 and Table 10 that this method largely underestimates the unit cell volume, in comparison to the DFT-D methods. Therefore, we used the initial volume as estimated by USPEX for the CSP simulations (Table 1, Table 3, Table 6 and Table 9). Figure 7 displays the volume vs. energy distributions of the predicted structures. β-HMX (Table 1 and Table 2, Simulations 8a and 8b) was found as the lowest energy structure (CxC = 0.991 in Simulation 8b) after the rotational mutation of a structure 10 meV/atom higher, which would have been discarded by USPEX if it had appeared at a late generation. For RDX (Table 3 and Table 4), Simulations 6a and 6b both found the best structure 0.75 meV/atom lower than the optimized reference of α-RDX, appearing as a slightly denser version of α-RDX, but with a CxC of 0.612 (relative orientation of the RDX molecules slightly altered). Interestingly, α-RDX was found two generations later, after the rotational mutation of this denser version (Figure 7b), with a CxC of 0.833. These two structures further optimized at a higher level of theory (CP2K PP) both yielded α-RDX with CxC = 0.999. Consequently, the denser version found with xTB has no physical relevance or meaning, since it transforms to α-RDX using CP2K PP. CL-20 (Table 6 and Table 7) was a tough case for the higher levels of theory, and xTB almost failed to find ε-CL20. Simulations 10a and 10b found a structure 6.11 meV/atom lower in SG 14 but with the molecules close to the ζ-conformation. Further optimization of this structure at a higher level of theory (CP2K PP) moved it up 1.73 meV/atom above ε-CL20. No structure with the molecules in ε-conformation and in SG 14 was retained among the good or best ones. Nonetheless, ε-CL20 (with CxC = 0.805) appeared at the 18th generation, too late and too high in energy, and was thus discarded by USPEX (Figure 7c). This structure reoptimized with CP2K PP yielded ε-CL20 with CxC = 0.999. xTB also failed to find either α-FOX-7 or β-FOX-7 as the lowest energy structures (Table 9 and Table 10). Simulation 4a found a wavy structure in SG 14 but with the C–C bond of the molecules significantly twisted, and 7.79 meV/atom lower than α-FOX-7. Further optimization of this structure with CP2K AE moved it up 4.49 meV/atom above α-FOX-7. Yet, α-FOX-7 or β-FOX-7 were found but at higher energy (Figure 7d), with α-FOX-7 surprisingly coming from the rotational mutation of the lowest energy structure. Simulation 4b interestingly yielded the structure in SG 33 already found in Simulations 1a, 3b, and 3c, but 6.13 meV/atom lower than α-FOX-7, and hence not as low as the “best” structure of Simulation 4a. Further optimization of this SG 33 structure with CP2K AE confirmed its stability and yielded the same energy as in Simulations 3b and 3c. Since it was found multiple times and by various methods, this FOX-7 structure in SG 33 could be given further attention.

### 7.2. How about a Hybrid xTB/DFT-D Approach?

The xTB method alone is not accurate enough for CSP. Nonetheless, we must emphasize that although the expected structures were rarely the lowest energy ones, they were yet all found at some point (Figure 7). We showed that further optimization using CP2K PP or CP2K AE can restore the energy scaling, making a hybrid xTB/DFT-D approach relevant. Table 11 shows that for systems involving 150+ atoms, computer resources could be saved by a factor of 20, which could make CSP affordable for crystals involving large molecules and cocrystals involving 200+ atoms. A hybrid xTB/DFT-D approach will be put to the test in a subsequent study involving cocrystals.

## 8. Conclusions

We used DFT-D to assess the capabilities of the EA embedded in USPEX for the CSP of famous and diversely challenging high-energy molecular crystals: HMX, RDX, CL-20, and FOX-7. Providing the EA with the experimental conformations of the molecules re-discovered the experimental structures. However, we also succeeded when we started from naïve, flat or neutral, initial conformations of the molecules, which is a more realistic process in the computational design of unknown molecular crystals, where experimental knowledge may obviously be missing. By doing so, and using fully flexible molecules in fully variable unit cells, we show that the experimental structures can be predicted in fewer than 20 generations. Nonetheless, we must emphasize that some molecular crystals such as CL-20 have naturally hindered evolutions, requiring as many attempts as space groups of interest to predict their structures, and some others such as FOX-7 may require the accuracy of all-electron calculations for a correct energy scaling of the predicted structures.

At these levels of theory (DFT-D), the CSP of high-energy molecular crystals was successful, but the process required significant computer resources. Using a lower level of theory such as xTB, we showed that the expected structures were all found, but not correctly ranked in energy. A hybrid xTB/DFT-D approach could thus be considered, potentially saving resources by a factor of 20, which will be the topic of a subsequent study involving large molecules and cocrystals.

The present study being an assessment of the CSP method and a display of various challenges, we mainly re-discovered known structures. The latent question is about the confidence one can have in the predictive capabilities of uncharacterized molecular crystals. The answer may be that if the same structure is found using different methods, then there must be something true about it. We have the example of the structure of FOX-7 in SG 33, structurally close to α-FOX-7 (SG 14 with β angle close to 90°), but never experimentally characterized, and yet found by USPEX using either QE (PP) or CP2K (AE or even xTB). Three methods agree with each other, and therefore further consideration could be given to this new FOX-7 phase.

## Figures and Tables

**Table 1:** USPEX parameters for HMX (the default initial volume estimated by USPEX is 432 Å^3^). The last column displays the generation number (#) at which β-HMX was found. The flat configuration is displayed in Figure 1. See the Supplementary Materials for USPEX input files of Simulations 1b, 5b, 7, and 8b.

	Initial Vol. (Å^3^)	SG for Random	USPEX Evolutionary Parameters					β-HMX Found at Gen. #
			Heredity	Random from SG	Soft Mut.	Rotation	Lattice Mut.	
**QE**								
1a	432 (def.)	14	0.1	0.1	0.2	0.4	0.2	1
1b	432 (def.)	Common	0.1	0.1	0.2	0.4	0.2	2
2a	570	14	0.1	0.1	0.2	0.4	0.2	6
2b	570	Common	0.1	0.1	0.2	0.4	0.2	14
3a	570	4	0.2	0	0.2	0.4	0.2	2
3b	570	4	0.2	0	0.2	0.4	0.2	2
3c	570	4	0.2	0	0.2	0.4	0.2	3
4a	520	14	0.1	0.1	0.2	0.4	0.2	3
4b	520	Common	0.1	0.1	0.2	0.4	0.2	5
5a	520 flat	14	0.1	0.1	0.2	0.4	0.2	9
5b	520 flat	Common	0.1	0.1	0.2	0.4	0.2	2
**CP2K**								
6a	520	14	0.1	0.1	0.2	0.4	0.2	1
6b	520	Common	0.1	0.1	0.2	0.4	0.2	12
7	520 flat	Common	0.1	0.1	0.2	0.4	0.2	2
**xTB**								
8a	432 flat	14	0	0.4	0.2	0.4	0	6
8b	432 flat	Common	0	0.4	0.2	0.4	0	3

**Table 2:** Reference structures (optimized experimental β-HMX) and β-HMX as discovered by USPEX starting from the flat molecule and the common SGs (after a few generations as “kept best”). The number in parenthesis refers to the Simulation ID in Table 1. The “P2_1_/a” lattice parameters from USPEX were switched to “P2_1_/n” and the axes were permutated for a straightforward comparison. The references and the best USPEX structures are in excellent agreement, as additionally displayed in Figure 2b for Simulation 7.

	a (Å)	b (Å)	c (Å)	α (°)	β (°)	γ (°)	V (Å^3^)	Space Group	E_tot_ (eV/atom)
QE alone	6.530	10.822	7.367	90.00	102.35	90.00	508.569	14	−230.3176
USPEX + QE (4b)	6.538	10.841	6.538	90.00	102.51	90.00	509.539	14	−230.3176
CP2K alone	6.545	10.919	7.335	90.00	102.80	90.00	511.105	14	−251.6243
USPEX + CP2K (7)	6.542	10.909	7.328	90.00	102.68	90.00	510.266	14	−251.6243
xTB alone	6.228	10.031	7.384	90.00	101.78	90.00	451.592	14	−73.3768
USPEX + xTB (8b)	6.228	10.028	7.386	90.00	101.78	90.00	451.636	14	−73.3768

**Table 3:** USPEX parameters for RDX (the default initial volume estimated by USPEX is 1298 Å^3^). The last column displays the generation number (#) at which α-RDX was found. The flat configuration is displayed in Figure 1.

	Initial Vol. (Å^3^)	SG for Random	USPEX Evolutionary Parameters					α-RDX Found at Gen. #
			Heredity	Random from SG	Soft Mut.	Rotation	Lattice Mut.	
**QE**								
1a	1298 (def.)	61	0.1	0.1	0.2	0.4	0.2	1
1b	1640	61	0.1	0.1	0.2	0.4	0.2	1
2a	1298 (def.)	Common	0.1	0.1	0.2	0.4	0.2	3
2b	1640	Common	0.1	0.1	0.2	0.4	0.2	1
3	1640 flat	Common	0.1	0.1	0.2	0.4	0.2	4
**CP2K**								
4	1640	Common	0.1	0.1	0.2	0.4	0.2	2
5	1640 flat	Common	0.1	0.1	0.2	0.4	0.2	3
**xTB**								
6a	1298 flat	61	0	0.4	0.2	0.4	0	1
6b	1298 flat	Common	0	0.4	0.2	0.4	0	2

**Table 4:** Reference structures (optimized experimental α-RDX) and α-RDX as discovered by USPEX starting from the flat molecule and the common SGs (after a few generations as “kept best”). The number in parenthesis refers to the Simulation ID in Table 3.

	A (Å)	b (Å)	c (Å)	α (°)	β (°)	γ (°)	V (Å^3^)	Space Group	E_tot_ (eV/atom)
QE alone	13.232	11.396	10.698	90.00	90.00	90.00	1613.172	61	−230.3134
USPEX + QE (3)	13.246	11.396	10.689	89.96	90.09	89.87	1613.413	61	−230.3132
CP2K alone	13.251	11.434	10.723	90.00	90.00	90.00	1624.791	61	−251.6209
USPEX + CP2K (5)	13.248	11.427	10.728	90.00	90.00	90.00	1624.087	61	−251.6209
xTB alone	12.850	10.767	10.205	90.00	90.00	90.00	1411.888	61	−73.3771
USPEX + xTB (6b)	12.847	10.683	10.130	90.00	90.00	90.00	1390.382	61	−73.3779

**Table 5:** Energy and volume of the CL-20 polymorphs calculated from variable-cell optimization using PBE-D. All PPs use NLCC. The lowest energies are in boldface. ε-CL20 is the most stable form for QE (with PPs). For CP2K, PPs rank γ-CL20 as very slightly more stable than ε-CL20, whereas ε-CL20 is the most stable form for AE.

CL-20	Exp. Amb. Conditions	QE PP	QE PP	CP2K PP	CP2K PP	CP2K AE	CP2K AE
		GBRV 1.5 D2 50 Ry	PSlib 1.0.0 D2 90 Ry	GTH- mDZVP-SR D2 600 Ry	GTH- mDZVP D2 600 Ry	DZVP D3(BJ) 600 Ry	6-311G** D3(BJ) 600 Ry
**E (meV/atom)**							
ε		**0**	**0**	0	0	**0**	**0**
γ		+0.19	+0.20	**−0.05**	**−0.15**	+0.78	+2.01
β		+0.90	+0.97	+0.66	+0.70	+1.13	+1.97
**V (Å^3^)**							
ε	1424.146	1433.032	1457.523	1448.528	1460.138	1442.687	1407.928
γ	1518.886	1521.047	1543.233	1533.996	1544.774	1533.098	1492.080
β	1465.981	1460.221	1485.979	1475.071	1487.424	1472.267	1440.647
**CxC**							
β vs. ε				0.720			
γ vs. ε				0.728			
β vs. γ				0.737			

**Table 6:** USPEX parameters for CL-20 (the default initial volume is 1179 Å^3^). All the simulations started with CL-20 molecules in the neutral conformation displayed in Figure 1. γ-CL20 was never found as the most stable form. So, the last column displays the generation number (#) at which ε-CL20 was found. QE uses GBRV 1.4 PP. CP2K uses the GHT-mDZVP-SR (PP) or 6-311G** level of theory (AE).

	Initial Vol. (Å^3^)	SG for Random	USPEX Evolutionary Parameters					ε-CL20 Found at Gen. #
			Heredity	Random from SG	Soft Mut.	Rotation	Lattice Mut.	
**QE**								
1	1179 (def.) neutral	14	0	0.4	0.2	0.4	0	15
2a	1420 neutral	14	0	0.4	0.2	0.4	0	2
2b	1420 neutral	Common	0	0.4	0.2	0.4	0	-
3	1420 neutral	19	0.25	0	0.25	0.25	0.25	-
**CP2K**								
4a (PP)	1420 neutral	14	0	0.4	0.2	0.4	0	8
4b (PP)	1420 neutral	Common	0	0.4	0.2	0.4	0	-
5 (PP)	1420 neutral	19	0.25	0	0.25	0.25	0.25	-
6 (PP)	1420 neutral	14, 19	0	0.4	0.2	0.4	0	-
7a (AE)	1420 neutral	14	0	0.4	0.2	0.4	0	1
7b (AE)	1420 neutral	Common	0	0.4	0.2	0.4	0	-
8 (AE)	1420 neutral	14, 19	0	0.4	0.2	0.4	0	-
**xTB**								
10a	1179 neutral	14	0	0.4	0.2	0.4	0	-
10b	1179 neutral	Common	0	0.4	0.2	0.4	0	-

**Table 7:** Reference structures (experimental ε-, γ-, and β-CL20 as optimized using various PBE-D levels of theory), and CL-20 crystals as discovered by USPEX starting from the flat molecule and various SGs (after a few generations as “kept best”). The number in parenthesis refers to the Simulation ID in Table 6.

**(A) QE + GBRV PP**									
**CL-20**	**a (Å)**	**b (Å)**	**c (Å)**	**α (°)**	**β (°)**	**γ (°)**	**V (Å^3^)**	**Space group**	**E_tot_ (meV/atom)**
**QE alone**									
ε	8.896	12.562	13.353	90.00	106.20	90.00	1433.032	14	0
γ	13.192	8.253	14.757	90.00	108.79	90.00	1521.047	14	+0.19
β	9.626	13.184	11.506	90.00	90.00	90.00	1460.221	29	+0.90
**USPEX + QE**									
(2a)	8.898	12.555	13.346	89.87	**106.18**	90.03	1431.964	**14**	+0.05
(2b)	8.852	12.650	13.082	90.00	**90.00**	90.00	1464.865	**19**	+0.92
**(** **B) CP2K + hybrid GTH-mDZVP-SR**									
	**a (Å)**	**b (Å)**	**c (Å)**	**α (°)**	**β (°)**	**γ (°)**	**V (Å^3^)**	**Space group**	**E_tot_ (meV/atom)**
**CP2K alone**									
ε	8.925	12.622	13.398	90.00	106.297	90.00	1448.528	14	0
γ	13.190	8.300	14.816	90.00	108.947	90.00	1533.996	14	−0.05
β	9.642	13.255	11.541	90.00	90.00	90.00	1475.071	29	+0.66
**USPEX + CP2K**									
(4a)	8.921	12.614	13.387	90.00	**106.21**	90.07	1446.614	**14**	−0.03
(4b)	8.894	12.639	13.137	90.00	**90.00**	90.00	1476.769	**19**	+0.87
(6)	13.247	8.813	13.020	90.00	91.20	90.00	1519.752	7	+0.09
(C) CP2K + AE 6-311G**									
	**a (Å)**	**b (Å)**	**c (Å)**	**α (°)**	**β (°)**	**γ (°)**	**V (Å^3^)**	**Space group**	**E_tot_ (meV/atom)**
**CP2K alone**									
ε	8.799	12.495	13.366	90.00	106.647	90.00	1407.928	14	0
γ	13.049	8.180	14.755	90.00	108.665	90.00	1492.080	14	+2.01
β	9.678	13.082	11.379	90.00	90.00	90.00	1440.647	29	+1.97
**USPEX + CP2K**									
(7a)	8.800	12.489	13.368	90.00	**106.622**	90.00	1407.627	**14**	−0.00
(8)	8.781	12.450	13.053	90.00	**90.00**	90.00	1427.030	**19**	+0.92
**(D) CP2K + GFN1-xTB**									
	**a (Å)**	**b (Å)**	**c (Å)**	**α (°)**	**β (°)**	**γ (°)**	**V (Å^3^)**	**Space group**	**E_tot_ (meV/atom)**
**xTB alone**									
ε	8.602	11.872	12.797	90.00	105.919	90.00	1256.695	14	0
γ	12.691	7.765	14.057	90.00	105.103	90.00	1337.267	14	+2.19
β	9.230	12.096	11.512	90.00	90.00	90.00	1285.266	29	+4.59
**USPEX + xTB**									
(10a&b)	12.544	7.101	13.941	90.00	90.00	87.282	1240.368	14	−6.11

(**A**) (2b), (**B**) (4b) and (**C**) (8): Despite in SG 19 and different β-angle, the CL-20 molecules are in ε-conformation.

**Table 8:** Energy and volume of α- and β-FOX-7 calculated from variable-cell optimization using PBE-D. All PPs use NLCC. The lowest energies are in boldface. The absolute energy differences are barely larger than 0.1 meV/atom for PP methods. Only the AE methods unambiguously rank α-FOX-7 as the most stable form.

FOX-7	Exp. Amb. Conditions	QE	QE	CP2K	CP2K	CP2K	CP2K
		GBRV 1.4 D2 50 Ry	PSlib 1.0.0 D2 90 Ry	GTH- mDZVP-SR D2 600 Ry	GTH- mDZVP D2 600 Ry	AE DZVP D3(BJ) 600 Ry	AE 6-311G** D3(BJ) 600 Ry
**E (meV/atom)**							
α		0	**0**	**0**	0	**0**	**0**
β		**−0.09**	+0.12	+0.01	**−0.06**	+0.91	+1.89
**V (Å^3^)**							
α	519.470	507.265	513.614	507.249	511.681	512.884	502.698
β	538.943	514.987	520.842	516.439	520.505	522.947	511.591
**CxC**							
β vs. α							0.496

**Table 9:** USPEX parameters for FOX-7 (the default initial volume is 433 Å^3^). All the simulations started with FOX-7 molecules in the flat conformation displayed in Figure 1. The last column displays the generation number (#) at which α, β, or another “best” polymorph was found. QE uses GBRV 1.5 PP. CP2K uses the GHT-mDZVP-SR (PP) or 6-311G** level of theory (AE).

	Initial Vol. (Å^3^)	SG for Random	USPEX Evolutionary Parameters					x-FOX-7 Found at Gen. #
			Heredity	Random from SG	Soft Mut.	Rotation	Lattice Mut.	
**QE**								
1a	520 flat	14	0	0.4	0.2	0.4	0	SG 33 at #7
1b	520 flat	Common	0	0.4	0.2	0.4	0	β at #2
**CP2K**								
2a	520 flat	14	0	0.4	0.2	0.4	0	-
2b	520 flat	Common	0	0.4	0.2	0.4	0	β at #3
3a (AE)	520 flat	14	0	0.4	0.2	0.4	0	α at #6
3b (AE)	520 flat	14	0	0.4	0.2	0.4	0	SG 33 at #1
3c (AE)	520 flat	Common	0	0.4	0.2	0.4	0	SG 33 at #3
3d (AE)	520 flat	Common	0	0.4	0.2	0.4	0	α at #5
**xTB**								
4a	433 flat	14	0	0.4	0.2	0.4	0	-
4b	433 flat	Common	0	0.4	0.2	0.4	0	SG33 at #2

**Table 10:** Reference structures (experimental α- and β-FOX-7 optimized using various PBE-D levels of theory), and FOX-7 crystals as discovered by USPEX starting from the flat molecule and various SGs (after a few generations as “kept best”). The number in parenthesis refers to the Simulation ID in Table 8.

**(A) QE + GBRV PP**									
**CL-20**	**a (Å)**	**b (Å)**	**c (Å)**	**α (°)**	**β (°)**	**γ (°)**	**V (Å^3^)**	**Space group**	**Etot (meV/atom)**
**QE alone**									
α	6.982	6.446	11.272	90.00	90.879	90.00	507.265	14	0
β	7.006	6.321	11.629	90.00	90.00	90.00	514.987	19	**−0.09**
**USPEX + QE**									
(1a)	6.576	7.001	11.194	90.00	90.00	89.70	516.020	33	+0.05
(1b)	7.008	6.313	11.629	90.00	90.00	90.00	514.477	19	−0.07
**(B) CP2K + hybrid GTH-mDZVP-SR**									
	**a (Å)**	**b (Å)**	**c (Å)**	**α (°)**	**β (°)**	**γ (°)**	**V (Å^3^)**	**Space group**	**Etot (meV/atom)**
**CP2K alone**									
α	6.981	6.464	11.243	90.00	90.895	90.00	507.249	14	**0**
β	7.004	6.345	11.622	90.00	90.00	90.00	516.439	19	+0.01
**USPEX + CP2K**									
(2a)	6.973	**6.863**	12.189	90.00	90.00	**60.60**	508.181	14	+1.62
(2b)	7.004	6.345	11.628	90.00	90.00	90.00	516.445	19	+0.01
**(** C) CP2K + AE 6-311G**									
	**a (Å)**	**b (Å)**	**c (Å)**	**α (°)**	**β (°)**	**γ (°)**	**V (Å^3^)**	**Space group**	**Etot (meV/atom)**
**CP2K alone**									
α	6.926	6.432	11.285	90.00	90.602	90.00	502.698	14	**0**
β	6.957	6.307	11.658	90.00	90.00	90.00	511.591	19	+1.89
**USPEX + CP2K**									
(3a)	6.925	6.434	11.274	90.10	90.598	90.30	502.328	14 α	+0.00
(3b)	6.941	6.435	11.368	90.00	90.00	89.76	507.730	33	+0.00
(3c)	6.941	6.437	11.364	90.00	90.00	90.00	507.773	33	+0.00
(3d)								14 α	+0.00
**(** **D) CP2K + GFN1-xTB**									
	**a (Å)**	**b (Å)**	**c (Å)**	**α (°)**	**β (°)**	**γ (°)**	**V (Å^3^)**	**Space group**	**Etot (meV/atom)**
**xTB alone**									
α	6.962	5.865	11.188	90.00	89.546	90.00	456.824	14	0
β	6.864	5.698	11.364	90.00	90.00	90.00	444.463	19	**−0.67**
**USPEX + xTB**									
(4a)	6.281	6.102	11.783	90.00	91.73	89.97	451.367	14 *	**−7.79**
(4b)	6.353	6.654	11.237	90.00	90.00	90.00	475.040	33	**−6.13**

* Significant C-C twist.

**Table 11:** Optimization time (CPU hours) used per generation (23 structures).

	Times (CPU Hours) for 1 Generation Using USPEX and …			
	QE	CP2K PP	CP2K AE	xTB
**HMX**	900	600	-	110 *
**RDX**	5000	3300	-	180 *
**CL-20**	7600	3400	23,400 *	170 *
**FOX-7**	1600	1500 *	2900 *	100 *

* Optimization in max 500 steps (instead of 250).

## Data Availability

The data that support the findings of this study are available within the article and its Supplementary Materials.

## Supplementary Materials

The following supporting information can be downloaded at: https://www.mdpi.com/article/10.3390/molecules28114471/s1, Supplementary Materials: Input files for USPEX to run HMX Simulations 1b, 5b, 7, and 8b (Table 1).
